# De Novo Assembly and Analysis of Tartary Buckwheat (*Fagopyrum tataricum* Garetn.) Transcriptome Discloses Key Regulators Involved in Salt-Stress Response

**DOI:** 10.3390/genes8100255

**Published:** 2017-10-03

**Authors:** Qi Wu, Xue Bai, Wei Zhao, Dabing Xiang, Yan Wan, Jun Yan, Liang Zou, Gang Zhao

**Affiliations:** 1 Key Laboratory of Coarse Cereal Processing, Ministry of Agriculture, Chengdu 610106, China; baixue@cdu.edu.cn (X.B.); xiangdabing@cdu.edu.cn (D.X.); wanyanyanbest@126.com (Y.W.); yanjun62@cdu.edu.cn (J.Y.); zouliang@cdu.edu.cn (L.Z.); 2 National Research and Development Center for Coarse Cereal Processing, Chengdu 610106, China; 3 College of Pharmacy and Biological Engineering, Chengdu University, Chengdu 610106, China; zhaowei6030@126.com

**Keywords:** *Fagopyrum tataricum*, salt stress, transcriptome, Illumina sequencing, expression analysis

## Abstract

Soil salinization has been a tremendous obstacle for agriculture production. The regulatory networks underlying salinity adaption in model plants have been extensively explored. However, limited understanding of the salt response mechanisms has hindered the planting and production in *Fagopyrum tataricum*, an economic and health-beneficial plant mainly distributing in southwest China. In this study, we performed physiological analysis and found that salt stress of 200 mM NaCl solution significantly affected the relative water content (RWC), electrolyte leakage (EL), malondialdehyde (MDA) content, peroxidase (POD) and superoxide dismutase (SOD) activities in tartary buckwheat seedlings. Further, we conducted transcriptome comparison between control and salt treatment to identify potential regulatory components involved in *F. tataricum* salt responses. A total of 53.15 million clean reads from control and salt-treated libraries were produced via an Illumina sequencing approach. Then we de novo assembled these reads into a transcriptome dataset containing 57,921 unigenes with N50 length of 1400 bp and total length of 44.5 Mb. A total of 36,688 unigenes could find matches in public databases. GO, KEGG and KOG classification suggested the enrichment of these unigenes in 56 sub-categories, 25 KOG, and 273 pathways, respectively. Comparison of the transcriptome expression patterns between control and salt treatment unveiled 455 differentially expressed genes (DEGs). Further, we found the genes encoding for protein kinases, phosphatases, heat shock proteins (HSPs), ATP-binding cassette (ABC) transporters, glutathione S-transferases (GSTs), abiotic-related transcription factors and circadian clock might be relevant to the salinity adaption of this species. Thus, this study offers an insight into salt tolerance mechanisms, and will serve as useful genetic information for tolerant elite breeding programs in future.

## 1. Introduction

Soil salinization has been an afflicting factor for plant agriculture [1]. More than 800 million hectares of land are suffering from salinization throughout the world [2]. Breeding and application of salt-tolerant elites for each food crop is becoming extremely urgent to feed the global population in future decades. Prior to achieving this goal there should be a good knowledge of the fundamental mechanisms of plant salt responses at the molecular, cellular, and whole plant levels. Recently, much progress was made especially in model plants (such as *Arabidopsis thaliana* and *Oryza sativa*), improving our understanding of this process. For salinity adaption, plants have generated a complex of strategies mainly containing osmotic tolerance, detoxification of ions, and tolerance of tissue to accumulated ions [2]. Thus, a series of signal perception, signal transduction, transcriptional regulation and biochemical reactions are employed to achieve the responses [3]. Protein kinases (e.g., calcium-dependent protein kinases (CDPKs) and CBL-interacting protein kinases (CIPKs)) are very important for hyperosmotic signal transduction to downstream gene transcription regulation [3,4]. Transmembrane transporters, such as H^+^-ATPase, Na^+^/H^+^ exchangers or Na^+^/H^+^ antiporters and Na^+^/K^+^ cation transporters, are critical for ion homeostasis [1,3]. Physiological evidences demonstrated accumulation of osmolytes (e.g., proline, glycine betaine and mannitol), activation of antioxidant enzymes (e.g., catalase, superoxide dismutase and peroxidase) were utilized to minimize the damages for plant cells [5]. Furthermore, plant hormones (e.g., abscisic acid (ABA), brassinosteroid (BR) and gibberellic acid (GA)) are highly associated with various stress signaling cascades and adaption [6]. Combination of genetic studies and transcriptome investigation has disclosed that many transcription factors are involved in salt stress responses [7,8,9,10,11,12,13,14,15,16,17,18]. MYB [9], dehydration-responsive element-binding proteins (DREB) [19], basic-domain leucine-zipper (bZIP) [20], CRT/DRE-binding factor (CBF) [21], WRKY [10,12], and NAC [22] were showed to regulate the stress-responsive genes. Nevertheless, understanding of the complexity of salt stress tolerance mechanism in plant deserves more in-depth knowledge.

Recently, next-generation sequencing (NGS) technologies have revolutionized the genomic and transcriptomic studies due to its high accuracy, throughput and sensitivity [23]. At present, NGS-based RNA sequencing (RNA-seq) has been widely employed to decipher various abiotic stress responses in many cases, especially for the species without validly annotated genome [24].

Tartary buckwheat (*Fagopyrum tataricum* Garetn.) is becoming highly attractive because of the high-quality proteins and pharmaceutical ingredients, such as rutin, quercetin and isoquercetin in the seeds [25]. In China, tartary buckwheat is mainly distributed in the marginal land of Sichuan province, Guizhou province and Yunnan province as the main food of the minorities [26]. However, in recent years, owing to water deficit and irrational irrigation in these arid areas, soil salinization has become a growing obstacle for tartary buckwheat planting. Therefore, dissecting the underlying salt stress mechanisms of *F. tataricum* and subsequent application is of great significance for protecting *F. tataricum* from yield loss attributing to soil salinization. Several genes related to salt response of buckwheat have been reported [27]. However, a comprehensive understanding of the salt adaption mechanism still remains limited in buckwheat.

In this study, we conducted a genome-wide transcriptome analysis of *F. tataricum* via an Illumina paired-end sequencing platform. Sequencing and annotation of the *F. tataricum* genome [28] was undergoing, thus we de novo assembled a transcriptome and carried out global expression profiling to identify the potential regulators involved in salt stress response. This study will provide a valuable source for updating our understanding of the salt tolerance regulatory network in *F. tataricum*.

## 2. Material and Methods

### 2.1. Plant Materials and Growth Conditions

Tatary buckwheat (*F. tataricum* cv. Chuanqiao No.1) was used as the experimental materials in this study. The seeds were provided by the National Research and Development Center for Coarse Cereal Processing, Chengdu, Sichuan Province, China. The seeds were sown and grown in small pots (12 cm in diameter and 10 cm in depth) at the density of 40 seeds for each pot. After germination, the seedlings were cultured in a growth chamber under controlled conditions: 23 °C, 12 h for dark, and 25 °C, 12 h for light with light intensity at 100 umol·^−2^·s^−1^. The humidity was about 65% with watering supplied every three days. After growth for two weeks, the seedlings were subjected to salinity treatment by supplying with 200 mM sodium chloride solution. Three independent biological replicates with eight seedling plants per replicate for each sample were collected at 0 h and 24 h after salinity treatment. The 0 h (control) and 24 h (salt treatment) samples were used for physiological analysis and total RNA extraction.

### 2.2. Physiological Analysis

The relative water content (RWC) was measured according to the method of Li et al. [29], and the electrolyte leakage (EL) was measured according to the protocol described by Ishitani [30]. The malondialdehyde (MDA) content, peroxidase (POD) and superoxide dismutase (SOD) activities were tested by the methods used in previous study [31].

### 2.3. RNA Extraction and cDNA Library Preparation

Total RNA of three control and three salt treated biological replicates was extracted using TaKaRa MiniBEST Universal RNA Extraction Kit (TaKaRa, Shiga, Japan) according to the manufacturer’s protocols. The integrity and quantity of total RNA samples were assessed by using 1% agarose gel electrophoresis, Qubit RNA HS Assay Kit (Thermo Fisher Scientific, Waltham, MA, USA) and 2100 Bioanalyzer RNA 6000 Nano Assay Kit (Agilent Technologies, Singapore). mRNA was isolated from 5 ug total RNA (RNA Integrity Number (RIN) > 7.0, pooled in equal quantity from the total RNA of three replicates) of each sample by magnetic oligo(dT) beads, fragmented into short sequences, and then subjected to complementary DNA (cDNA) library construction by using Illumina TrueSeq RNA library method according to TrueSeq RNA Sample Preparation guide (Illumina Technologies, San Diego, CA, USA).

### 2.4. Illumina Sequencing and Sequences Assembly

The cDNA libraries of control and salt treatment samples were sequenced using Illumina Hiseq 2500 platform (Illumina Technologies) to generate 150 bp paired-end reads. The unqualified reads, including (1) adaptors contaminated reads; (2) unknown nucleotides more than 5% reads and (3) reads of which more than 50% of bases showed a *q*-value less than 5, were removed from the raw reads to obtain clean reads. Trinity program [32] was used to de novo assemble high-quality clean reads into transcriptome. The longest sequences of redundant and chimeric transcripts were defined as unigenes, and then were subjected to downstream functional annotation and coding sequence (CDS) prediction.

### 2.5. Coding Sequence Prediction, Functional Annotation and Classification of Unigenes

For CDS prediction, unigenes were compared with GenBank NR (NCBI non-redundant protein) [33] and Swiss-Prot databases [34] by BLAST [35] (E-value cut-off = 1 × 10^−5^), and the best alignment was used to determine the 5′-3′ orientation and the putative coding sequence region. ESTScan 3.0.3 [36] was used to predict the putative open reading frame (ORF) region of the unigenes without alignment in GenBank NR and Swiss-Prot databases.

To annotate the unigenes dataset, the sequences were aligned against GenBank NR [33], GenBank NT (NCBI nucleotide sequences) [33], Swiss-Prot databases [34] and KOG (euKaryotic Orthologous Groups) [37] using NCBI BLAST 2.2.28+ [35] with an E-value threshold of 1×10^-5^. Meanwhile, these unigenes were also annotated by KEGG (Kyoto Encyclopedia of Genes and Genomes) [38] using KAAS (E-value cut-off = 1 × 10^−10^) [39], by GO (Gene Ontology) [40] using Blast2GO v2.5 (E-value cut-off =1 × 10^−6^) [41] and by Pfam (Protein family) [42] using HMMER 3.0 [43] (E-value cut-off = 0.01).

### 2.6. Identification of Transcription Factor Families

To identify the potential transcription factor encoding genes in the transcriptome data, the assembled unigenes sequences were analyzed using PlantTFcat online tool (http://plantgrn.noble.org/PlantTFcat) as previously described [40].

### 2.7. Expression Analysis

The clean reads were mapped onto the assembled unigenes transcriptome using Bowtie [44], and the read count number for each gene was measured by RNA-Seq by Expectation-Maximization (RSEM) [45]. The estimated expression levels of all genes were calculated and normalized to fragments per kilobase of transcript per killion fragments mapped (FPKM) [46]. To identify the differentially expressed genes (DEGs), read count for each gene was normalized with the Trimmed mean of M-values (TMM) method [47], and then DEGseq [48] was used to screen the genes whose *q*-value were less than 0.005 and |log2FoldChange| > 1 between control and salinity treatment samples. Further, the identified DEGs were subjected to GO enrichment analysis by GOseq based on the Wallenius non-central hypergeometric distribution with the corrected *p*-value less than 0.05 [49]. Moreover, KEGG pathway enrichment analysis of the DEGs was also implemented using KEGG Orthology Based Annotation System (KOBAS) [50].

### 2.8. Identification of Simple Sequence Repeats (SSRs)

MISA (MIcroSAtellite) V1.0 [51] with default parameters was used to analyze distribution of various types of SSRs in all *F. tataricum* unigenes. The minimum number of repeat units for mono-nucleotide and di-nucleotide repeats were ten and six, respectively, while that for tri-nucleotide, tetra-nucleotide, penta-nucleotide and hexa-nucleotide repeats was five.

### 2.9. Reverse Transcription and Real-Time PCR

Two micrograms of total RNA for each sample were subjected to DNA digestion and reverse transcription to synthesize first-strand cDNA using EasyScript One-Step gDNA Removal and cDNA Synthesis SuperMix Kit (Transgen Biotech, Beijing, China). The cDNA reaction product was diluted 10-fold and 2 uL of the dilution was used to carry out real-time PCR using TransStart Green qPCR SuperMix (Transgen Biotech) on a real-time PCR system (LineGene 9600, BIOER, Hangzhou, China). The real-time PCR program consists of 94 °C for 5 min, 39 cycles of denaturing at 95 °C for 10 s, annealing and extension at 59 °C for 20 s. An actin gene was used as the internal control [52]. The relative expression level of detected genes was quantified by the 2^−ΔΔCt^ algorithm [37] using Microsoft Excel 2015 (Microsoft, Redmond, WA, USA). For each gene, we performed at least three biological replicates to determine the relative expression level. The related primers used for real-time PCR were list in Table S8.

### 2.10. Statistical Analysis

**Figure 1:**
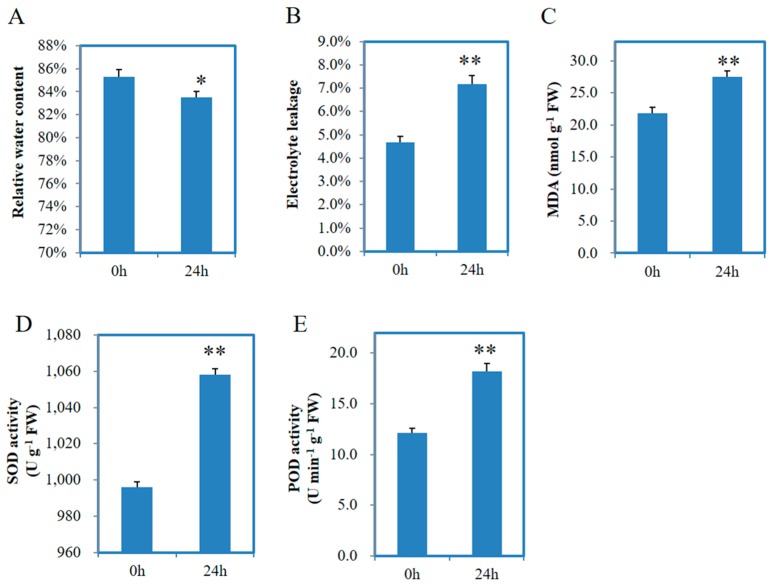
Measurement of relative water content (**A**); electrolyte leakage (**B**); malondialdehyde (MDA) (**C**); superoxide dismutase (SOD) (**D**) and peroxidase (POD) activities (**E**) in tartary buckwheat seedlings under control and salt stress conditions. 0 h and 24 h represent control and salt treatment conditions. Values are mean ± SD (*n* = 3). Single asterisk (*) and double asterisks (**) stand for *p* < 0.05 and *p* < 0.01, respectively, determined by student’s *t*-test.

The data of various physiological indices in Figure 1 were subjected to Student’s *t*-test analysis using Microsoft Excel 2015.

## 3. Results

### 3.1. Physiological Analysis

In order to evaluate the effects of salt on physiological activities in *F. tataricum* seedlings, we measured a series of key physiological indices including relative water content (RWC), electrolyte leakage (EL), malondialdehyde (MDA) content, superoxide dismutase (SOD) and peroxidase (POD) activities. Two-week-old Chuan Qiao No.1 seedlings were treated with 200 mM NaCl solution, and then the aerial parts of seedling plants were collected at 0 h (control) and 24 h (salt treatment) after salt treatment. The collected seedling samples were used for physiological analysis.

As shown in Figure 1A, the RWC in salt stress plants was decreased (*p*-value < 0.05) than that in control plants. The EL analysis demonstrated that the EL was significantly increased (*p*-value < 0.01) from 4.7% to 7.2% after salt treatment (Figure 1B). The MDA content was sharply increased (*p*-value < 0.01) by 25.9% after salt treatment than control (Figure 1C). Meanwhile, we found both SOD and POD activities were significantly increased (*p*-value < 0.01) under salt stress (Figure 1D,E). These results suggest that salt stress of 200 mM NaCl solution is able to significantly affect the physiological activities in *F. tataricum* seedlings.

### 3.2. RNA-Seq and De Novo Assembly of the F. tataricum Transcriptome

To elucidate the underlying salt stress responsive mechanisms in tartary buckwheat, we carried out RNA-seq analysis. Total RNA of control and salt treated samples were extracted, followed by cDNA library construction and paired-end Illumina deep sequencing. After removing the low-quality reads and adaptor sequences from reads, in total we obtain 23.81 million and 29.34 million clean reads from control and salt treatment libraries, which yielded 7.14 billion and 8.80 billion nucleotides, respectively (Table 1). With respect to GC content, control library reached 48.76% and that of salt treatment library accounted for 46.70% (Table 1). The percentage of Q20 sequences (with an error probability of 0.01) in control library reached 97.85%, while that in salt treatment library was 98.03% (Table 1). Using Trinity platform [32], we de novo assembled the two libraries clean reads into 75,287 transcripts, from which 57,921 unigenes were generated (Table 1). All the unigenes length ranged from 201 bp to 18,866 bp. The mean length, median length and N50 length of all assembled unigenes were 768 bp, 387 bp, and 1400 bp, respectively (Table 1).

**Figure 2:**
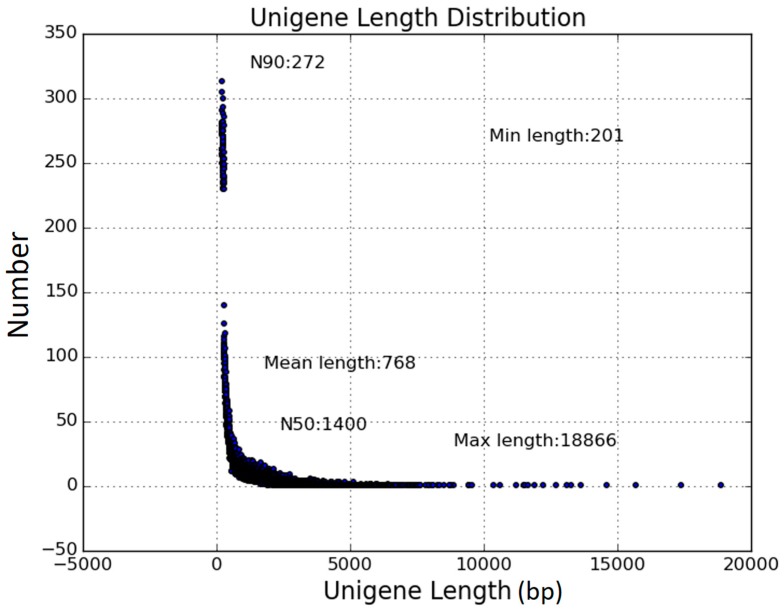
Length distribution of unigenes.

An overview of the unigenes size distribution was showed in Figure 2. Among these unigenes, 23,845 (41.17%) were longer than 500 bp, and that longer than 1000 bp reached 13,930 (24.05%). The assembled unigenes dataset was used as reference transcriptome for further analysis. The reference transcriptome data generated in this study could be found at the Gene Expression Omnibus (GEO), National Centre for Biotechnology Information (NCBI) (https://www.ncbi.nlm.nih.gov/geo/) under the accession number GSE104167.

### 3.3. Functional Annotation and Classification of Transcriptome Sequences

**Figure 3:**
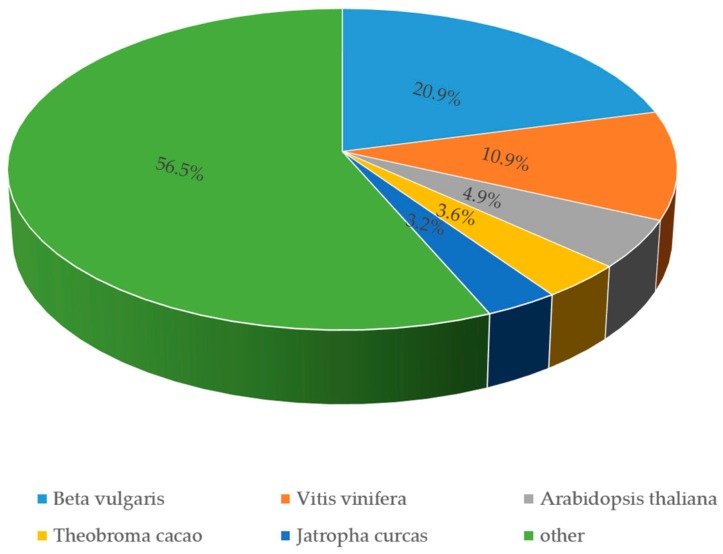
Species distribution of the matched transcriptome sequences in Genebank non redundant (NR) database.

In order to fully annotate the unigenes dataset, we aligned each unigene sequence against Genebank NR [33], Genebank NT [33], Pfam [42], KOG [37], Swiss-Prot [34], KEGG [38] and GO [40] databases. Our analysis showed that, out of 57,921 unigenes, 30,500 (52.65%) were revealed to have significant matches in Genebank NR (Table 2), displaying the highest hit rate among all annotation events in the six public databases. The annotated unigenes number in Pfam, Genebank NT and Swiss-Prot were 20,859 (36.01%), 21,064 (36.36%) and 23,445 (40.47%), respectively (Table 2). By using Blast2GO v2.5, we found that 20,834 (35.96%) matched known proteins in GO (Table 2). The hits similarity in KEGG and KOG were 11,206 (19.34%) and 11,791 (20.35%) (Table 2), respectively, indicating relatively lower annotation rate than that in the other four databases. Among all unigenes, 4813 (8.3%) could well match the known proteins in every database and 36,688 (63.34%) had significant matches in at least one database (Table 2). On the other hand, we found that those with non-annotation in any one of the databases reached 21,233 (36.66%) (Table 2), which could result from the specific genes in *F. tataricum*, or the error happened in sequencing. According to the similarity search in Genebank NR, over 20% of the *F. tataricum* transcriptome sequences shared strong similarity with genes of *Beta vulgaris* followed by *Vitis vinifera* (10.9%), *Arabidopsis thaliana* (4.9%), *Theobroma cacao* (3.6%), and *Jatropha curcas* (3.2%) (Figure 3).

**Figure 4:**
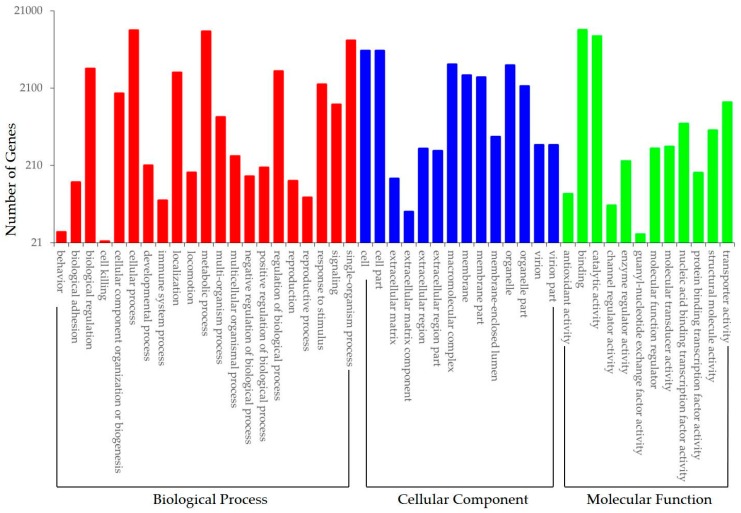
Gene ontology analysis of the *Fagopyrum tataricum* transcriptome.

To determine the gene ontology of transcriptome sequences, Blast2GO was used to analyze the predicted protein sequences similarity with Genebank NR and Pfam proteomes. We found 20,834 (35.96%) unigenes could be assigned to 56 GO sub-categories under biological process, cellular component and molecular function categories (Table S1). Among biological process, ‘cellular process’ (11,753, 56.4%) was overrepresented followed by ‘metabolic process’ (11,368, 54.6%), ‘single-organism process’ (8864, 42.5%), ‘biological regulation’ (3736, 17.9%), ‘regulation of biological process’ (3477, 16.7%), ‘localization’ (3334, 16%), and ‘response to stimulus’ (2345, 11.3%) (Figure 4). With respect to cellular component, both ‘cell’ and ‘cell part’ (6399, 30.7%) were the most dominant groups followed by ‘macromolecular complex’ (4258, 20.4%), ‘organelle’ (4150, 19.9%), ‘membrane’ (3068, 14.7%), and ‘membrane part’ (2889, 13.9%) (Figure 4). Under molecular function term, ‘binding’ (11,891, 57.1%), ‘catalytic activity’ (9823, 47.1%), ‘transporter activity’ (1374, 6.6%), ‘nucleic acid binding transcription factor activity’ (727, 3.5%), ‘structural molecule activity’ (601, 2.9%) and ‘molecular transducer activity’ (369, 1.8%) were highly represented (Figure 4).

**Figure 5:**
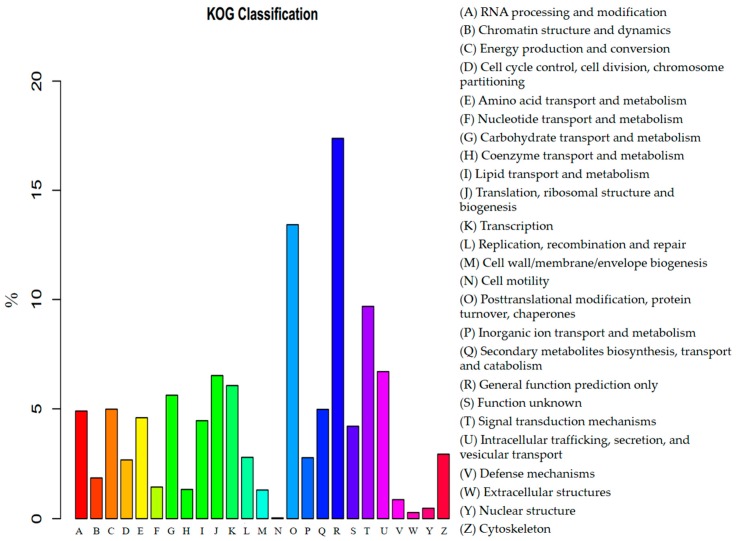
KOG (euKaryotic Orthologous Groups) function classification of the *F. tataricum* transcriptome.

Further, KOG classification revealed that 11,791 transcriptome sequences were clustered into 25 functional categories (Table S2). Among the 25 groups, ‘General function prediction only’ represented the most enriched term (2049, 17.4%), which was followed by ‘Posttranslational modification, protein turnover, chaperones’ (1583, 13.4%), ‘Signal transduction mechanisms’ (1142, 9.7%), ‘Intracellular trafficking, secretion, and vesicular transport’ (790, 6.7%), ‘Translation, ribosomal structure and biogenesis’ (769, 6.5%), ‘Carbohydrate transport and metabolism’ (663, 5.6%), ‘Energy production and conversion’ (588, 5.0%), ‘Secondary metabolites biosynthesis, transport and catabolism’ (587, 5.0%), ‘RNA processing and modification’ (578, 4.9%) (Figure 5). The smallest group was ‘Cell motility’ which only contained 4 genes.

**Figure 6:**
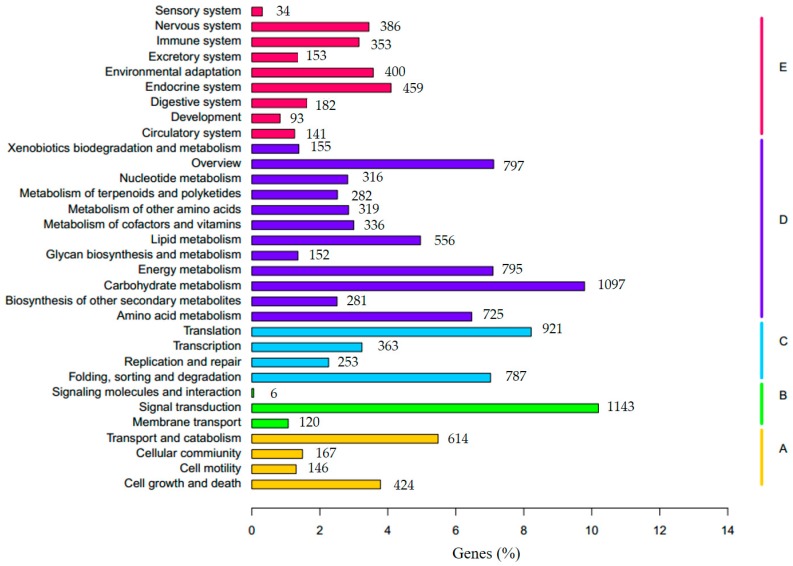
KEGG (Kyoto Encyclopedia of Genes and Genomes) classification of the *F. tataricum* transcriptome. (**A**–**E**) beside the right vertical lines means five different KEGG functional groups. (**A**) Cellular Processes; (**B**) Environmental Information Processing; (**C**) Genetic Information Processing; (**D**) Metabolism; (**E**) Organismal Systems.

To better understand the biological functions of our transcriptome genes on the biochemical pathways, we performed KEGG classification by comparing the unigenes with KEGG proteome data using the online KAAS. In total, we obtained 11,206 orthologs involved in 273 pathways in KEGG protein database (Table S3). The most enriched pathways were ‘Carbon metabolism’ (ko01200), ‘Biosynthesis of amino acids’ (ko01230), ‘Ribosome’ (ko03010), ‘Plant hormone signal transduction’ (ko04075), and ‘Protein processing in endoplasmic reticulum’ (ko04141). All the annotated unigenes could be categorized into five KEGG functional groups, out of which ‘Metabolism’ was overrepresented (5811, 51.9%) (Figure 6). In ‘Metabolism’, ‘Carbon metabolism’ (1079, 9.6%) was highly enriched followed by ‘Overview’ (797, 7.1%), ‘Energy metabolism’ (795, 7.1%), ‘Amino acid metabolism’ (725, 6.5%), and ‘Lipid metabolism’ (556, 5.0%) (Figure 6). 2324 unigenes were classified into ‘Genetic Information Processing’, including ‘Translation’ (921, 8.2%), ‘Folding, sorting and degradation’ (787, 7.0%), ‘Transcription’ (363, 3.2%), and ‘Replication and repair’ (253, 2.3%) (Figure 6). In ‘Organismal Systems’, 2201 unigenes were mainly distributed in ‘Endocrine system’ (459, 4.1%), ‘Environmental adaptation’ (400, 3.6%), and other sub-categories. In addition, 1,351 unigenes were involved in ‘Cellular Processes’, in which ‘Transport and catabolism’ (614, 5.5%), ‘Cell growth and death’ (424, 3.8%), ‘Cellular community’ (167, 1.5%), and ‘Cell motility’ (146, 1.3%) were highly enriched (Figure 6). ‘Environmental Information Processing’ accounted for 1269 unigenes, consisted of ‘Signal transduction’ (1143, 10.2%), ‘Membrane transport’ (120, 1.1%), and ‘Signaling molecules and interaction’ (6, 0.05%) (Figure 6). Together, these results suggest carbohydrate and amino acid metabolism, various signal transduction and translation events become active in *F. tataricum* under salt stress.

### 3.4. Identification of Transcription Factor Families

In plants, transcription factors usually play pivotal roles in growth and development processes. Accumulating evidence indicates that many transcription factor families are involved in abiotic stress responses [53]. We predicted the potential transcription factors using PlantTFcat online tool. In total, we identified 93 transcription factor families, including 3107 unigenes, accounting for 5.4% of the transcriptome (Table S4). Out of 93 families, ‘C2H2’ (473, 15.2%) was mostly overrepresented, followed by ‘WD40-like’ (285, 9.2%), ‘MYB-HB-like’ (193, 6.2%), CCHC (176, 5.7%), ‘PHD’ (148, 4.8%), ‘bHLH’ (121, 3.9%), ‘AP2-EREBP’ (104, 3.4%), and ‘bZIP’ (95, 3.1%).

### 3.5. Analysis of Differentially Expressed Genes

To investigate the DEGs between control and salt treatment libraries, we mapped the clean reads from each sample onto the *F. tataricum* reference transcriptome using Bowtie. Approximately 81.3% and 84.7% of the total high-quality reads from control and salt treatment libraries were mapped to the reference transcriptome, respectively. Using DEGseq, with the cutoff of *q*-value < 0.005 and |log2FoldChange|>1, we obtained 455 DEGs (Figure S1). Among these DEGs, 404 unigenes had higher expression levels under salt treated conditions, whereas only 51 unigenes had higher expression levels under control conditions.

**Figure 7:**
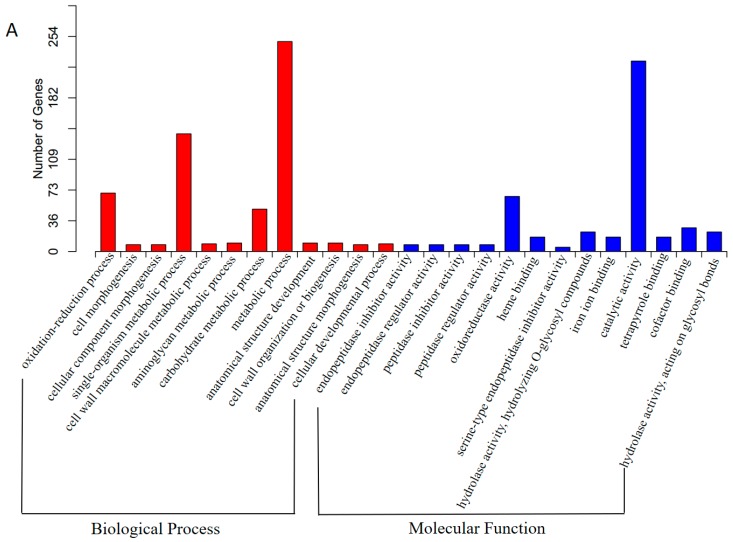

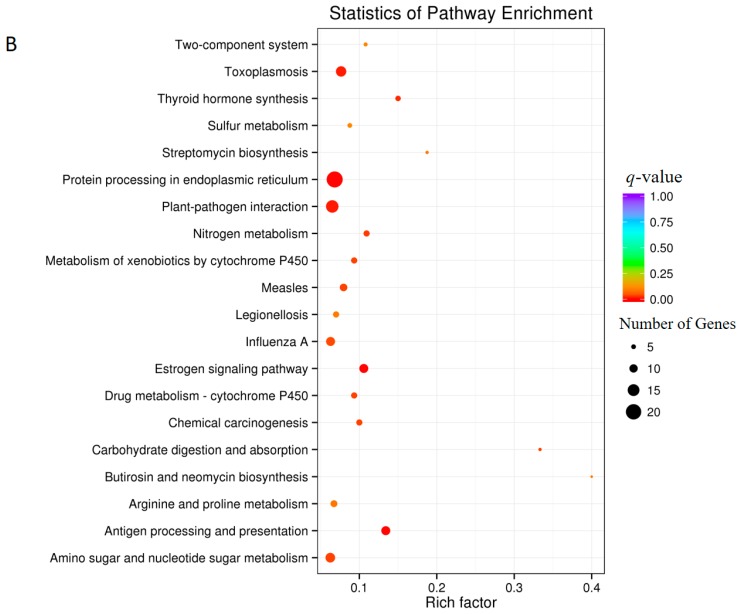
Gene ontology and KEGG analysis of differentially expressed genes (DEGs). (**A**) GO (gene ontology) classification of the DEGs. BP: GO term type of biological process; MF: GO term type of molecular function; (**B**) KEGG classification of the DEGs.

To understand the biological function of the DEGs, we performed GO term enrichment using Blast2GO. Out of 455 DEGs, 363 DEGs were highly abundant in 25 GO terms (Corrected *p*-Value < 0.05). GO enrichment analysis revealed that these DEGs were overrepresented in ‘oxidation-reduction process’, ‘endopeptidase inhibitor activity’, ‘endopeptidase regulator activity’, ‘peptidase inhibitor activity’, ‘peptidase regulator activity’, ‘cell morphogenesis’, and ‘cellular component morphogenesis’ (Figure 7A and Table S5). Most of the up-regulated DEGs in salt treatment were enriched in ‘cell morphogenesis’, ‘cellular component morphogenesis’, ‘oxidation-reduction process’, ‘endopeptidase inhibitor activity’, ‘endopeptidase regulator activity’, ‘peptidase inhibitor activity’, ‘peptidase regulator activity’, ‘cell wall macromolecule metabolic process’, and ‘oxidoreductase activity’ (Table S5). Meanwhile, the down-regulated DEGs under salt treated conditions were overrepresented in ‘ATP metabolic process’, ‘purine nucleoside triphosphate metabolic process’, ‘ribonucleoside triphosphate metabolic process’, ‘purine ribonucleoside triphosphate metabolic process’, ‘nucleoside triphosphate metabolic process’ and ‘monovalent inorganic cation transport’ (Table S5). These results indicated the biological functions described above might be activated under salt stress in *F. tataricum*.

In addition, in order to further predict which biochemical pathways these DEGs may be involved in, we carried out KEGG enrichment analysis. The results revealed that the DEGs were highly represented in pathway terms ‘Antigen processing and presentation’ (ko04612), ‘Protein processing in endoplasmic reticulum’ (ko04141), ‘Estrogen signaling pathway’ (ko04915), ‘Toxoplasmosis’ (ko05145), and ‘Plant-pathogen interaction’ (ko04626) (Figure 7B and Table S6).

### 3.6. Validation of Differentially Expressed Genes by Real-Time PCR

**Figure 8:**
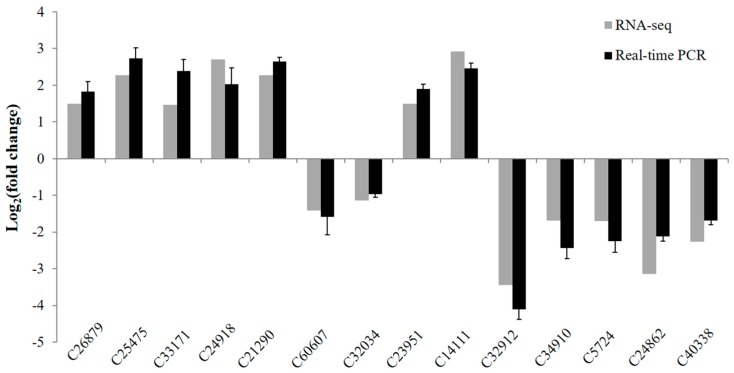
Validation of RNA sequencing (RNA-seq) expression profiles by real-time PCR. The gray bars stand for fold changes based on read count number from normalized RNA-seq data. The black bars stand for fold changes of relative expression levels that were obtained by real-time PCR based on the 2^−ΔΔCt^ algorithm. Values are mean ± SD (*n* = 3).

To confirm the reliability of expression profile data obtained by RNA-seq analysis, we conducted real-time PCR analysis. Fourteen unigenes randomly selected from the DEGs were further qualified the expression levels in control and salt treated samples. The fold changes of these genes obtained by real-time PCR were consistent with that obtained by RNA-seq analysis (according to Pearson correlation analysis, R^2^ = 0.9372) (Figure 8), thus confirming the accuracy and strong reliability of the expression analysis results.

### 3.7. Identification of Simple Sequence Repeats 

Molecular markers, such as Random Amplified Polymorphic DNA (RAPD), Restriction Fragment Length Polymorphism (RFLP), Single Nucleotide Polymorphism (SNP) and Simple Sequence Repeats (SSRs), have been extensively used as powerful tools for genetic study and marker-assisted selection (MAS) in many food crops [54]. In this study, we performed SSRs screening using MISA search tool [51].

**Figure 9:**
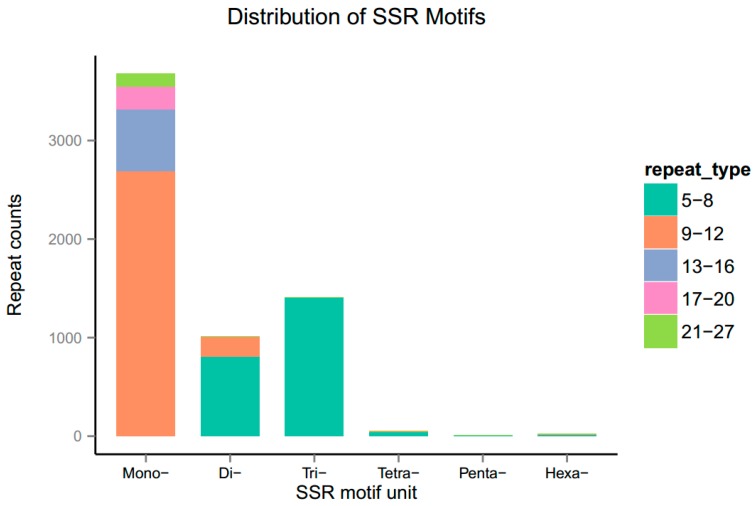
Distribution of simple sequence repeats (SSRs) identified from *F. tataricum* unigenes.

By screening 57,921 transcriptome sequences, a total of 6176 potential SSRs were identified to distribute among 5455 unigenes, out of which 599 unigenes were found to harbor more than one SSR. The most abundant SSR type was mononucleotide repeats (3678, 59.6%), followed by tri-nucleotide (1410, 22.8%), di-nucleotide (1010, 16.4%), tetra-nucleotide (49, 0.8%), hexa-nucleotide (21, 0.3%), and penta-nucleotide repeats (8, 0.1%) (Figure 9). Among all SSRs, the most dominant motif was A/T (3641, 59%), in which 9–12 tandem repeats were overrepresented followed by 13–16, 17–20 and 21–27 tandem repeats. In di-nucleotide repeats, AT/AT displayed the higher frequency (545, 8.8%) followed by AG/CT (378, 6.1%). For tri-nucleotide repeats, AAG/CTT showed the most abundant occurrence (434, 7.0%) followed by ATC/ATG (218, 3.5%), ACC/GGT (181, 2.9%), AAC/GTT (125, 2.0%), and AGG/CCT (120, 1.9%) (Table S7). Among di-nucleotide and tri-nucleotide repeats, SSRs with 5–8 tandem repeats were the most common tandem repeat.

## 4. Discussion

Soil salinization is a severe threat to the yield of many crops. Recently, transcriptome studies on a large number of species, such as rice, *Arabidopsis*, smooth cordgrass (*Spartina alterniflora*) [14], wheat (*Triticum aestivum*) [15] and *Caragana korshinskii* [55], have been intensively carried out to decode the salt stress response. These studies have largely improved our understanding of salt-responsive mechanisms of plants. However, more investigation is needed especially for the uncharacterized species. In this study, we characterized the salt-responsive transcriptome of *F. tataricum*. To our knowledge, before this study, there are seldom reports focused on globally deciphering the salt response via RNA-seq in *F. tataricum*.

Chuan Qiao No. 1 is one of the most widely grown tartary buckwheat cultivar in Sichuan Province. Considering the importance of seedling stage for tartary buckwheat development and yield, we used two-week-old seedlings as the experimental materials for physiological activities analysis and RNA-seq analysis. By measuring the RWC, we found salt stress could cause water loss in seedling plants at 24 h after salt treatment. EL and MDA content are usually used to evaluate the integrity of plant cell membranes under abiotic stress [56]. Significantly increased EL and MDA content indicated 200 mM NaCl solution has largely affected the robust status of cell membrane in tartary buckwheat seedlings. When exposed to abiotic stress, plant antioxidant enzymes such as POD and SOD are usually activated to protect cells and subcellular systems from damage caused by reactive oxygen species (ROS) [57]. Consistently, we tested and found increased POD and SOD activities in seedling plants after salt treatment for 24 h.

Due to unavailable genome information, we de novo assembled a transcriptome of *F. tataricum*. A total of 53.15 million clean reads yielded 57,921 unigenes with N50 length of 1400 bp and total length of 44.5 Mb. Recently Zhu et al. generated an aluminum responsive transcriptome containing 39,815 contigs [58], whereas Logacheva et al. assembled a floral transcriptome containing 25,401 contigs [59] in *F. tataricum*. Thus, our high-throughput RNA-seq yielded considerably more genes. We speculated the gaps could result from the differences between salt stress and aluminum stress processes, or from the sequencing approaches.

Among all transcriptome sequences, 36,688 (63.34%) unigenes were annotated in public databases. Through GO classification, 20,834 (35.96%) unigenes were categorized into 56 sub-categories, out of which ‘metabolic process’, ‘binding’, ‘cellular process’ and ‘catalytic activity’ were strongly represented. Meanwhile, KEGG and KOG classification were used as alternative approaches to map the unigenes to biological pathways. The results showed that metabolic pathways of ‘Posttranslational modification, protein turnover, chaperones’, ‘Signal transduction mechanisms’, ‘Intracellular trafficking, secretion, and vesicular transport’ in KOG and ‘Signal transduction’, ‘Carbohydrate metabolism’ and ‘Translation’ in KEGG were overrepresented. These results can supply with important cues for dissecting the molecular mechanisms of salt responses in *F. tataricum*.

Through comparison of gene expression patterns between control and salt treatment, we obtained 455 DEGs. This is relatively a smaller number compared with the DEGs numbers obtained in *Arabidopsis* [60,61], wheat [15], and rice [62]. We speculated that this gap could result from the fact that our RNA-seq data mainly reflected the early-stage gene expression profiles under salt stress (24 h after salt treatment). Anyway, among these DEGs, the genes related to protein kinases, phosphatases, HSPs, ABC transporters, GSTs, abiotic-related transcription factors and circadian clock were dominant. Protein kinases and phosphatases act as key components in plant stress signaling [2]. Previous studies indicated that the mitogen-activated protein kinases (MAPK) pathways were tightly related to salt stress response [18,63,64,65]. Among our DEGs, *c33984_g1* and *c34545_g1*, two homologs of MAPK kinase, were up-regulated after salinity treatment (Table 3), suggesting MAPK signaling pathways are also involved in buckwheat salt signaling. Meanwhile, four serine-threonine protein kinase encoding genes, namely *c1037_g1*, *c25455_g1*, *c33021_g2*, *c25455_g1* and *c22689_g1* showed significantly higher expression levels in salt treatment (Table 3), which was consistent with the observation in *Spartina alterniflora* salt stress transcriptome data [14]. In *Arabidopsis thaliana*, calcium signaling is elicited by salt stress, and calcium-dependent protein kinases (CDPKs) and CBL-interacting protein kinases (CIPKs) mediate the calcium signal and downstream responses [1,4,66]. It is worth noting that *c38839_g1*, *c31052_g1*, *c60566_g1* (three putative CDPKs) and *c49233_g1* (a putative CIPK) were up-regulated under salt stress (Table 3). Previous study showed that overexpression of a *L*-type lectin-domain-containing receptor kinase (AtLPK1) enhanced the salt-stress tolerance in *Arabidopsis* [67]. Coincidentally, our analysis showed that the expression of *c10866_g1* (a homolog of *AtLPK1*) was increased by about 9-fold in salt treatment than in control (Table 3). Besides, one putative protein phosphatase 2C (PP2C) gene (*c38748_g1*) and one tyrosine-protein phosphatase gene (*c60922_g1*) were significantly induced by salt stress (Table 3), suggesting protein phosphatases may play important roles in *F. tataricum* under stress.

In *Arabidopsis* and creeping bentgrass (*Agrostis stolonifera* L.), heat shock proteins (HSPs) function as protein chaperones to attenuate plant abiotic stress responses [68,69,70]. A lot of potential HSPs homologs identified by our study, such as *c10992_g1*, *c22339_g1*, *c26417_g3*, *c29031_g1*, *c29386_g1*, *c29386_g2*, *c30033_g1*, *c31857_g1*, *c32499_g1*, *c32766_g1*, *c35678_g1*, *c38902_g1* and *c54547_g1*, were shown to accumulate more transcripts under salt stress (Table 3), suggesting the involvement of HSPs regulatory mechanisms. ATP-binding cassette (ABC) transporters are also correlated with salt and other abiotic stress resistance [71,72]. Overexpression of *AtABCG36* displayed higher fresh weight and stronger drought and salt resistance than wild type in *Arabidopsis*. Here, two ABC transporter B family members (*c39646_g1* and *c30623_g2*) and two ABC transporter C family members (*c38901_g1* and *c33388_g1*) were significantly up-regulated (Table 3), suggesting ABC transporters might share similar function in *F. tataricum* as well as in *Arabidopsis*. Glutathione S-transferases (GSTs) play vital roles in response to the oxidative stress including drought, salt and other stress [73,74,75]. Elevated expression levels of *GST*s in tobacco and soybean were proven to enhance salt stress tolerance [74,75]. In our study, five putative *GST* encoding genes, namely *c39155_g1*, *c39970_g1*, *c4684_g2*, *c59876_g1* and *c60552_g1*, showed significantly higher expression levels under salinity stress (Table 3), indicating GSTs might also impact the stress tolerance in *F. tataricum*.

Brassinosteroid (BR) metabolism participates in regulating plant growth and abiotic stress resistance [56]. Genetic and biochemical evidences demonstrated that somatic embryogenesis receptor kinases (SERKs) interacted with brassinosteroid insensitive 1 (BRI1) and were indispensable for early steps in BR signaling [76]. In rice (*Oryza sativa*), interaction between 14-3-3 proteins and OsBZR1 was essential for maintaining robust BR responses [77]. Notably, the fact that *c25693_g1* (putative SERK1) and *c39300_g1* (predicted 14-3-3) were remarkably up-regulated under salt treated conditions (Table 3) indicated that BR signaling might also participate in salt stress signaling pathways in *F. tataricum*.

Using the PlantTFcat online tool, 3,107 out of 57,921 unigenes were predicted to encode for various putative transcription factors, which belong to 93 families. Interestingly, a great deal of abiotic stress responsive transcription factors, such as WD40-like, MYB, apetala2/ ethylene-responsive element-binding protein (AP2/EREBP), WRKY, bZIP and C2C2-Dof, were strongly dominant. Notably, many of them showed differentially expression patterns between control and salt treated conditions. In this study, four *AP2/EREBP* members (*c21290_g1*, *c33171_g2*, *c44576_g1* and *c50089_g1*), one *MYB* member (*c23951_g1*), eight *NAC* members (*c14111_g2*, *c15464_g1*, *c24596_g2*, *c26879_g1*, *c44171_g1*, *c44256_g1*, *c60319_g1* and *c8889_g1*), seven *WRKY* members (*c10876_g1*, *c23849_g1*, *c25475_g1*, *c28962_g2*, *c45063_g1*, *c49337_g1* and *c49928_g1*) and three *ZAT* members (*c38942_g1*, *c51237_g1* and *c61192_g1*) were increased by more than 2 folds (7.4 > log_2_.fold changes >1.2) in salt treatment than control samples (Table 3). Accumulating evidence demonstrates that *AP2*/*EREBP*, *MYB*, *NAC*, *WRKY* and *ZAT* family members are closely associated with abiotic stress defense [1,2,56,57,78]. Overexpression of *AP2*/*EREBP* resulted in improved salt tolerance in rice and *Arabidopsis* [79]. In *MYB* family, *OsMYB3R*-2 and *OsMYB48-1* overexpressors have been shown to harbor increased tolerance to cold, drought and salt stress in plant [9,13]. In soybean (*Glycine max*) and rice, NAC transcription factors were found to confer resistance to drought and salt stress [7,11,80]. Incorporation of wheat (*Triticum aestivum*) *WRKY* genes (*TaWRKY2* and *TaWRKY19*) and soybean (*GmWRKY13*, *GmWRKY21* and *GmWRKY54*) into *Arabidopsis* plants led to increased abiotic stress tolerance [10,12]. Mutation in *ZAT7* abolished the salt tolerance, whereas elevated expression or RNA interference (RNAi) of *ZAT10* gave rise to more tolerance to osmotic and salinity stress in *Arabidopsis* [8,81]. This indicates these transcription factors may confer the salinity adaption in *F. tataricum*, as well.

Circadian clock coordinates the plant growth and development in response to environment changes [82,83]. Many microarray and metabolite-profiling studies revealed that plant circadian clock controlled stress-related genes modulate the abiotic responses [84]. Mutation of Gigantea (GI) in *Arabidopsis* resulted in enhanced salt tolerance, which is due to the release of SOS2 kinase [85]. In addition, the rice osgi mutant exhibited increased osmotic stress and harbored higher abundance of *OsDREB1E*, *OsAP37*, *OsAP59*, *OsLIP9*, *OsLEA3*, *OsRAB16A*, and *OsSalT* [86]. Consistently, in our transcriptome data, *c32034_g1* (predicted *FtGIGANTEA* in tartary buckwheat) was decreased by more than 2-fold under salt conditions (Table 3). This finding indicates that in *FtGIGANTEA* may also function in salt-stress adaption.

Taken together, we de novo generated and annotated a salt responsive transcriptome of *F. tataricum*. Genome-wide assay of the transcriptional differences between control and salt-treated conditions led to identification of a lot of key regulators in salt response mechanisms. Consequently, the present study could further improve our understanding of the salt tolerance mechanisms in *F. tataricum*. The transcriptome data could supply valuable molecular information for future plant breeding strategies.

## Figures and Tables

**Table 1:** Characteristics of generated read data and assembled *Fagopyrum tataricum* transcriptome data.

**Item**	**CQ_0h**	**CQ_Na24h**
Raw reads number	23,828,556	29,351,455
Clean reads	23,816,967	29,340,188
Base number (Gb)	7.14	8.80
GC content (%)	48.76%	46.70%
Q20 percentage	97.85%	98.03%
**Unigenes**		
Number of unigenes	57,921	
Min length of unigenes (bp)	201	
Mean length of unigenes (bp)	768	
Max length of unigenes (bp)	18,866	
N50 of unigenes (bp)	1,400	
Total Nucleotides of unigenes (bp)	44,508,371	

CQ_0h and CQ_Na24h represent control and salt-treated samples respectively.

**Table 2:** Summary of functional annotation in six public databases.

	Number of Unigenes	%
Annotated in Genebank NR	30,500	52.65
Annotated in Genebank NT	21,064	36.36
Annotated in KEGG	11,206	19.34
Annotated in SwissProt	23,445	40.47
Annotated in Pfam	20,859	36.01
Annotated in GO	20,834	35.96
Annotated in KOG	11,791	20.35
Annotated in all databases	4813	8.3
Annotated in at least one database	36,688	63.34
Not annotated in all database	21,233	36.66
Total Unigenes	57,921	100

**Table 3:** **Key regulators involved in salt stress responses in *F. tataricum*.** CQ_0h and CQ_Na24h represent control and salt-treated samples respectively.

Gene_ID	CQ_Na24_Readcount	CQ_0_Readcount	log2.Fold_Change	*p*−Value	*q*−Value	Annotation
MAPK						
*c33984_g1*	149.2283698	47.67405994	1.6462	1.10E−14	1.77E−12	Mitogen-activated protein kinase 9-like (*Nelumbo nucifera*)
*c34545_g1*	59.3142507	5.238326231	3.5012	1.99E−13	2.75E−11	Mitogen-activated protein kinase 2-like (*Citrus sinensis*)
**Serine−threonine protein kinase**						
*c1037_g1*	36.96338405	1.587371585	4.5414	3.94E−10	3.99E−08	probable serine-threonine protein kinase (*Beta vulgaris* subsp. *vulgaris*)
*c25455_g1*	32.83888317	1.957758288	4.0681	9.25E−09	7.77E−07	serine-threonine protein kinase (*Ricinus communis*)
*c33021_g2*	18.93342307	1.005335337	4.2352	1.04E−05	0.0005562	serine-threonine protein kinase (*Ricinus communis*)
*c25455_g1*	32.83888317	1.957758288	4.0681	9.25E−09	7.77E−07	serine-threonine protein kinase (*Ricinus communis*)
*c22689_g1*	45.015981	15.55624154	1.5329	5.30E−05	0.0024396	serine/threonine protein kinase (*Eucalyptus grandis*)
**CDPKs**						
*c38839_g1*	35.23502178	8.042682698	2.1313	9.55E−06	0.00051234	Calcium-dependent protein kinase 19 (*Theobroma cacao*)
*c31052_g1*	232.8033275	105.0294992	1.1483	8.13E−14	1.17E−11	Calcium-dependent protein kinase 26 isoform X1 (*Vitis vinifera*)
*c60566_g1*	41.9127851	14.39216904	1.5421	8.99E−05	0.0039211	Calcium-dependent protein kinase 11 (*Beta vulgaris* subsp. *vulgaris*)
**CIPKs**						
*c49233_g1*	703.207759	280.4356467	1.3263	3.54E−47	2.54E−44	CBL-interacting serine/threonine-protein kinase 4-like (*Beta vulgaris* subsp. *vulgaris*)
**LPK**						
*c10866_g1*	25.21837679	2.751444081	3.1962	3.82E−06	0.00022042	kinase, putative (*Ricinus communis*)
**PP2C**						
*c38748_g1*	173.1111939	63.54777579	1.4458	2.75E−14	4.10E−12	protein phosphatase 2C 6 (*Morus notabilis*)
**PTPase**						
*c60922_g1*	38.06325095	6.137836796	2.6326	2.23E−07	1.57E−05	tyrosine-protein phosphatase (*Beta vulgaris* subsp. *vulgaris*)
**HSPs**						
*c10992_g1*	595.8528933	171.2244817	1.7991	2.80E−60	2.80E−57	heat shock protein 70 (*Ageratina adenophora*)
*c22339_g1*	128.9593941	50.9017155	1.3411	4.77E−10	4.76E−08	heat shock protein, putative (*Ricinus communis*)
*c26417_g3*	280.7410263	129.0003975	1.1219	7.62E−16	1.36E−13	Heat shock cognate 70 kDa protein (*Glycine soja*)
*c29031_g1*	123.4207786	52.065788	1.2452	7.83E−09	6.64E−07	hypothetical protein B456_013G139100 (*Gossypium raimondii*)
*c29386_g1*	1492.912194	346.1528304	2.1086	1.92E−180	1.06E−176	heat shock protein 83 (*Beta vulgaris* subsp. *vulgaris*)
*c29386_g2*	864.9274743	402.3986969	1.104	2.04E−44	1.37E−41	heat shock protein 90-2 (*Populus euphratica*)
*c30033_g1*	222.2909568	78.20450677	1.5071	6.84E−19	1.60E−16	heat shock protein 70 (*Gossypium hirsutum*)
*c31857_g1*	196.9940181	78.25741915	1.3319	1.90E−14	2.88E−12	class I heat shock protein-like (*Nelumbo nucifera*)
*c32499_g1*	674.21841	200.4321188	1.7501	1.27E−65	1.50E−62	heat shock protein 2 (*Sesamum indicum*)
*c32766_g1*	93.5279675	46.50998745	1.0079	1.78E−05	0.00091511	heat shock 70 kDa protein 14-like (*Vitis vinifera*)
*c35678_g1*	57.62516939	7.354821678	2.9699	1.37E−11	1.59E−09	class II heat shock protein-like (*Populus euphratica*)
*c38902_g1*	275.9880301	87.94038582	1.65	6.68E−26	2.40E−23	class I heat shock protein (*Vitis vinifera*)
*c54547_g1*	852.1611621	267.4721121	1.6717	1.06E−77	1.40E−74	heat shock protein (*Solanum lycopersicum*)
**ABC Transporter**						
*c39646_g1*	110.6151854	44.97552825	1.2983	1.77E−08	1.44E−06	ABC transporter B family member 25 (*Vitis vinifera*)
*c30623_g2*	110.5759045	22.06446503	2.3252	1.56E−16	2.92E−14	ABC transporter B family member 11-like (*Beta vulgaris* subsp. *vulgaris*)
*c38901_g1*	526.9933692	238.2115626	1.1455	3.36E−29	1.32E−26	ABC transporter C family member 4 (*Gossypium arboreum*)
*c33388_g1*	55.58255943	10.10626576	2.4594	1.58E−09	1.45E−07	ABC transporter C family member 3-like (*Pyrus* x *bretschneideri*)
**GST**						
*c39155_g1*	250.1804389	40.74253735	2.6184	5.03E−40	2.97E−37	glutathione S-transferase (*Eucalyptus grandis*)
*c39970_g1*	45.17310484	12.32858598	1.8735	3.69E−06	0.00021425	glutathione transferase (*Populus trichocarpa*)
*c4684_g2*	107.5119895	14.60381858	2.8801	8.78E−20	2.30E−17	glutathione S-transferase (*Eucalyptus grandis*)
*c59876_g1*	188.4307686	61.59001751	1.6133	1.09E−17	2.21E−15	glutathione S-transferase (*Citrus sinensis*)
*c60552_g1*	79.93675509	7.19608452	3.4736	1.79E−17	3.55E−15	glutathione S-transferase U17-like (*Citrus sinensis*)
**SERK1**						
*c25693_g1*	21.29028072	0.634948634	5.0674	1.37E−06	8.58E−05	somatic embryogenesis receptor kinase 1-like (*Zea mays*)
**14-3-3**						
*c39300_g1*	82.49001753	39.10225338	1.077	2.27E−05	0.0011344	14-3-3 protein, putative (*Ricinus communis*)
**AP2/EREBP**						
*c21290_g1*	39.55592746	8.201419857	2.2699	1.13E−06	7.13E−05	ethylene−responsive transcription factor (*Sesamum indicum*)
*c33171_g2*	52.40080161	18.94263425	1.468	2.32E−05	0.0011555	AP2-like ethylene−responsive transcription factor AIL5 (*Eucalyptus grandis*)
*c44576_g1*	67.79893822	28.67851331	1.2413	1.97E−05	0.00099714	ethylene-responsive transcription factor ABR1-like (*Populus euphratica*)
*c50089_g1*	84.41478461	14.23343188	2.5682	2.66E−14	3.99E−12	putative ethylene response factor 5 (*Vitis aestivalis*)
**MYB**						
*c23951_g1*	55.8182452	19.84214482	1.4922	9.98E−06	0.00053404	transcription factor MYB108 (*Beta vulgaris* subsp. *vulgaris*)
**NAC**						
*c14111_g2*	46.90146711	6.190749182	2.9214	1.42E−09	1.32E−07	NAC domain-containing protein (*Boehmeria nivea*)
*c15464_g1*	48.47270554	0.846598179	5.8394	2.27E−13	3.13E−11	NAC transcription factor (*Fagus sylvatica*)
*c24596_g2*	89.3249047	35.76877305	1.3204	2.99E−07	2.07E−05	NAC domain-containing protein 2-like (*Tarenaya hassleriana*)
*c26879_g1*	85.8288992	30.53044682	1.4912	4.36E−08	3.41E−06	NAC domain-containing protein 78 (*Vitis vinifera*)
*c44171_g1*	65.59920442	29.63093626	1.1466	7.51E−05	0.0033343	NAC domain protein (*Medicago truncatula*)
*c44256_g1*	232.5040066	87.56999912	1.4087	4.75E−18	1.01E−15	NAC transcription factor (*Fagus sylvatica*)
*c60319_g1*	54.52197349	8.99510565	2.5996	7.35E−10	7.10E−08	NAC domain-containing protein (*Boehmeria nivea*)
*c8889_g1*	97.37750165	31.27122023	1.6388	4.90E−10	4.87E−08	NAC domain-containing protein 1 (*Salvia miltiorrhiza*)
**WRKY**						
*c10876_g1*	37.67044134	2.222320219	4.0833	7.35E−10	7.10E−08	WRKY transcription factor 31 (*Nicotiana sylvestris*)
*c23849_g1*	215.1811029	69.89726214	1.6222	4.05E−20	1.10E−17	WRKY protein (*Rheum australe*)
*c25475_g1*	64.77430424	13.3868337	2.2746	4.47E−10	4.47E−08	WRKY4 transcription factor (*Vitis aestivalis*)
*c28962_g2*	179.0819	55.55800548	1.6886	7.33E−18	1.52E−15	WRKY transcription factor 33 (*Glycine max*)
*c45063_g1*	30.24633976	3.439305101	3.1366	5.17E−07	3.43E−05	WRKY transcription factor 75 (*Nelumbo nucifera*)
*c49337_g1*	37.04194597	4.762114756	2.9595	6.23E−08	4.76E−06	WRKY transcription factor 70-like (*Citrus sinensis*)
*c49928_g1*	18.1085229	0.105824772	7.4188	1.57E−05	0.00081139	WRKY transcription factor 73 (*Gossypium hirsutum*)
**ZAT**						
*c38942_g1*	96.94541108	21.85281549	2.1494	1.57E−13	2.22E−11	zinc finger protein ZAT10 (*Beta vulgaris* subsp. *vulgaris*)
*c51237_g1*	19.79760421	0.423299089	5.5475	2.80E−06	0.00016544	zinc finger protein ZAT11-like (*Solanum tuberosum*)
*c61192_g1*	52.32223969	6.08492441	3.1041	4.95E−11	5.54E−09	zinc finger protein ZAT10 (*Populus euphratica*)
**GIGANTEA**						
*c32034_g1*	202.2969478	445.575204	−1.1392	1.05E−19	2.73E−17	protein GIGANTEA (*Beta vulgaris* subsp. *vulgaris*)

MAPK: mitogen-activated protein kinase; CDPKs: calcium-dependent protein kinases; CIPKs: CBL-interacting serine/threonine-−protein kinases; LPK: L-type lectin-domain-containing receptor kinase; PP2C: protein phosphatase 2C; PTPase: tyrosine-protein phosphatase; HSPs: heat shock proteins; ABC Transporter: ATP-binding cassette Transporter; GST: glutathione S-transferase; SERK1: somatic embryogenesis receptor kinase 1; 14-3-3: 14-3-3 protein; AP2/EREBP: AP2-like ethylene−responsive transcription factor; MYB: MYB transcription factor; NAC: NAC transcription factor; WRKY: WRKY transcription factor; ZAT: zinc finger protein ZAT.

## Supplementary Materials

The following are available online at www.mdpi.com/2073-4425/8/10/255/s1. Figure S1: Numbers of up- and down-regulated genes between control and salt treatment in *Fagopyrum. tataricum*. Table S1: Gene ontology of the *F. tataricum* transcriptome at level2. Table S2: Statistics of euKaryotic Orthologous Groups (KOG) function classification of the *F. tataricum* transcriptome. Table S3: Enrichment of the *F. tataricum* transcriptome on Kyoto Encyclopedia of Genes and Genomes (KEGG) pathways at Hierarchy2. Table S4: Identification of the transcription factor families in *F. tataricum*. Table S5: Gene ontology of the differentially expressed genes (DEGs). Table S6: Enrichment of the DEGs on KEGG pathways. Table S7: Statistics of the frequency for different simple sequence repeats (SSRs) in *F. tataricum*. Table S8: The primers used for real-time PCR in this study.
