# Supplementary material for: De Novo Assembly and Analysis of Tartary Buckwheat (Fagopyrum tataricum Garetn.) Transcriptome Discloses Key Regulators Involved in Salt-Stress Response
Source: Genes (Basel). 2017 Oct 3;8(10):255. doi: 10.3390/genes8100255 (PMC5664105; doi:10.3390/genes8100255)
Supplement: Supplementary file 1 [file genes-08-00255-s001.zip › Supplementary table S8-real-time primers.docx]

| **Gene ID** | **Primer-f (5’-3’)** | **Primer-r (5’-3’)** |
| --- | --- | --- |
| q-*FeActin*  (*c24824_g1*) | ACCTTGCTGGACGTGACCTTAC | CCATCAGGAAGCTCATAGTTC |
| q-*c26879_g1* | CGGAATGTGTTGGTCTTTGG | TCTACGAGAAATTGCTGAGGTC |
| q-*c25475_g1* | TTCCTCACCCAACCACTATTG | AGTCGGATCCTTTGTCAACG |
| q-*c33171_g2* | GCTTCCCTCTAAACCCTAATCC | CGCCCAGAAAGTCCTCTAAC |
| q-*c24918_g1* | GATTTATGTTTTGGCTGGGAGG | AACTGAGGAACCATGTGCTAG |
| q-*c21290_g1* | CAACGGCGATCTGAAAACTG | CACTTTCTCACGATTCTCCCTC |
| q-*c60607_g1* | ACAAGCCATCCTGACCAAG | AACAATCTATAGCCCAACCCC |
| q-*c32034_g1* | ACATTCTCCAAAACCCACCG | TTCCCATGTCAAACACCTCTC |
| q-*c23951_g1* | TTCCGGTTCTCTTCAATCCTG | TCCAATACATAACTCGACACGG |
| q-*c14111_g2* | AGCTCACCATCAAATCAGACC | CTCTTCCAAAACCCTCCCAG |
| q-*c32912_g3* | GAAGCCAGTCGAAGATACCAG | ACCCCATCAATTAACCTTCCG |
| q-*c34910_g1* | ACCTTGCTTCATCCCAAATTTC | GGGAAGTGAAGGTGTTTTGTG |
| q-*c5724_g1* | CCGTCATAAACCAAAATCGTCTG | TCCTATCCACCTCCAAGTCC |
| q-*c24862_g1* | AACCTCAATCCAAACTAGCCC | TGGACGATGCTTGAGTTGTG |
| q-*c40338_g1* | GCCGGATACAAATCGAAAACG | AATCTCAGTGGCGTCTCAAC |

**Table S8. The primers used for real-time PCR in this study.**
