# Supplementary figures and images for: De Novo Assembly and Analysis of Tartary Buckwheat (Fagopyrum tataricum Garetn.) Transcriptome Discloses Key Regulators Involved in Salt-Stress Response
Source: Genes (Basel). 2017 Oct 3;8(10):255. doi: 10.3390/genes8100255 (PMC5664105; doi:10.3390/genes8100255)

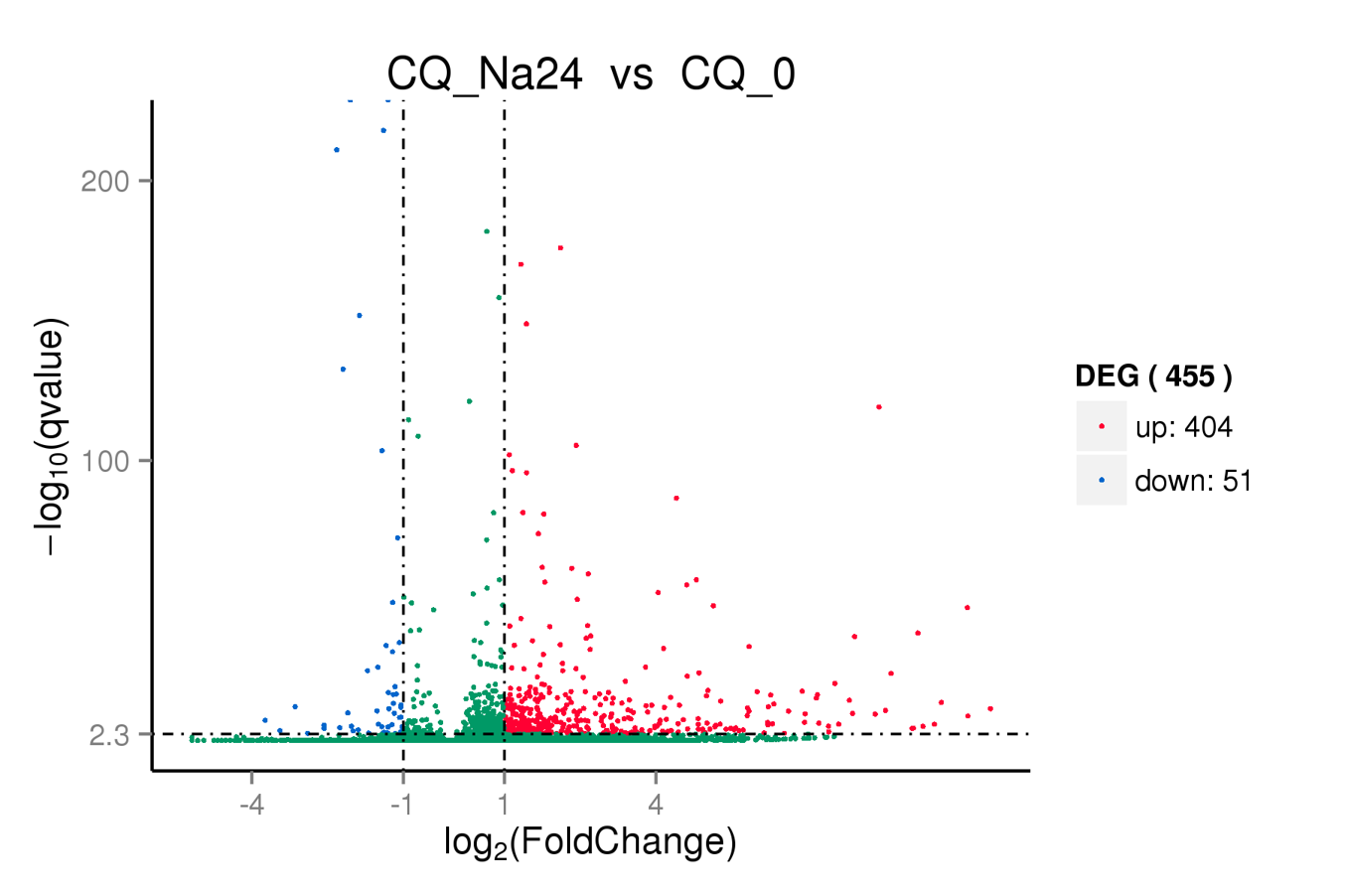

Supplement: Supplementary file 1 [file genes-08-00255-s001.zip › Supplementary figure S1.tif]
